# Cromolyn-mediated improvement of intestinal barrier function is associated with enhanced piglet performance after weaning

**DOI:** 10.1186/s12917-015-0588-1

**Published:** 2015-10-28

**Authors:** Alessandro Mereu, Gemma Tedó, Adam J. Moeser, Gerald Rimbach, Ignacio R. Ipharraguerre

**Affiliations:** Lucta S.A., Can Parellada 28, 08170 Montornés del Vallés, Barcelona Spain; Gastrointestinal Stress Biology Laboratory, Department of Large Animal Clinical Sciences, College of Veterinary Medicine, Michigan State University, East Lansing, 48824 MI USA; Institute of Human Nutrition and Food Science, University of Kiel, Hermann-Rodewald-Straße 6-8, D-24118 Kiel, Germany

**Keywords:** Weaning stress, Gut permeability, Mast cells, Pig

## Abstract

**Background:**

Previous work showed that weaning stress causes gut barrier dysfunction partly by triggering the release of corticotropin releasing factor (CRF) and thereby inducing the degranulation of intestinal mast cell (MC). This study investigated the hypothesis that attenuating the weaning-induced activation of the CRF-MC axis via administration of a MC stabilizing agent (cromolyn) may improve gut permeability and piglet performance after weaning.

**Results:**

To test the hypothesis twenty piglets were weaned (20 ± 1.0 d of age; 6.4 ± 0.4 kg of BW) and injected intraperitoneally with saline (control, *n* = 10) or 20 mg/kg BW of sodium cromolyn (cromolyn, *n* = 10) at – 0.5, 8 and 16 h relative to weaning. Piglets were housed individually and fed ad libitum a pre-starter diet from one to 15 d post-weaning followed by a starter diet until the end of the study on d 36. Cromolyn improved intestinal permeability as indicated by the reduced recovery of cobalt and mannitol in plasma samples. Cromolyn treated pigs consumed more feed (369 vs. 313 g/d; *P* < 0.009), gained more BW (283 vs. 238 g/d; *P* < 0.006), and grew more efficiently (0.60 vs. 0.40; *P* < 0.042) than their control counterparts. As a result, cromolyn treated pigs were 1.4 kg heavier than those in the control group by d 36 after weaning (16.5 vs. 17.9 kg; *P* < 0.002).

**Conclusions:**

In agreement with our hypothesis, present data indicate that the cromolyn-mediated improvement of intestinal permeability is associated with enhanced pig performance after weaning.

## Background

Mammals must constantly cope with the external environment to maintain homeostasis. To this end, they are provided with extended and complex selective surfaces like the gut mucosa. This epithelium, which is the largest mucosal surface in the body, plays the critical function of ensuring the absorption of nutrients while limiting the entrance of detrimental compounds such as toxins and bacteria that inhabit the luminal environment [1]. Over the last decade stress has been identified as a trigger of gastrointestinal dysfunctions through activation of the brain-gut axis. This mechanism entails the release of corticotropin-releasing factor (CRF), the main neuro-mediator of stress and a key effector of stress-related intestinal disturbances [2, 3]. In the gut, CRF binds to CRF_1_ and CRF_2_ receptors which are expressed in multiple effector cells in the gut including neurons, epithelial cells, and immune cells such as the mast cells (MC) serving thereby as end-effector of the brain-gut axis. Activation of MC by CRF induces their degranulation and release of several cytokines and proteases causing increased intestinal permeability through a mechanism that involves disruption of tight junctions [2, 4–6].

In most settings of pig production piglets are weaned at an early age (19 – 28 days of life), which expose them to several stressors like mother and littermate separation, transportation, dietary changes, and commingling with unfamiliar mates. This combination of stressful events compromises piglet welfare and health by resulting in transient anorexia, increased susceptibility to diarrhea and enteric infections, and reduced growth [7, 8]. Furthermore, neonatal maternal separation induces long-term impairment of the intestinal barrier function [2]. In turn, compromised gut permeability facilitates transmigration of endotoxins and other harmful compounds from the lumen into the lamina propria and from there into systemic circulation. Increased levels of circulating endotoxins trigger the release of pro-inflammatory cytokines causing a shift in the partitioning of energy from growth towards maintenance of the immune response, finally resulting in decreased animal performance [9, 10]. In line with this notion, it has been demonstrated that early weaning induces long term intestinal hyperpermeability in pigs [11] and that stabilization of MC with cromolyn, a drug that prevents MC degranulation trough a mechanism that appears to involve inhibition of the opening of Ca^2+^ channels [12, 13], can attenuate weaning-induced mucosal dysfunction [6].

Although compelling evidence indicates that treating pigs with cromolyn around weaning can improve intestinal barrier function, the impact of this treatment on animal performance after weaning has not been systematically investigated. The current study is based on the hypothesis that lessening the weaning-induced increase in gut permeability with cromolyn may improve piglet performance during the post-weaning phase. Therefore the aim of the present study was to investigate the effect of treating piglets with cromolyn immediately before and after weaning on gut barrier function and animal performance during the nursing phase.

## Methods

### Animals and housing

All experimental procedures were approved by the Laboratory Animal Care Advisory Committee of the Faculty of Veterinary Sciences of the Universitat Autónoma de Barcelona, Spain. Two days before weaning, twenty piglets (50:50 male:female ratio; Large White x Landrace × Piétrain) belonging to 12 different litters were selected and identified by ear-tag at the farm of origin (Cassá de la Selva, Spain). Piglets were weaned at 20 ± 1.0 d of age and 6.4 ± 0.42 kg of BW and immediately transported (50 km) to the nursery facility located at the Swine Experimental Unit of Lucta S.A. (Girona, Spain). At arrival, piglets were distributed into 20 individual pens (0.35 m^2^/pen) equipped with fully slatted plastic floors plus a nipple drinker and a feeder, offered ad libitum access to water and a non-medicated pre-starter diet from d one (weaning) to d 15 and a non-medicated starter diet from d 16 to d 36 of experiment (Table 1). Individual BW was measured at weaning (initial) and then weekly until d 36 after weaning. Feed consumption was measured weekly from weaning until d 36 after weaning. However, feed consumption on week 5 (d 30 to 36) was excluded from statistical analysis because of technical problems that did not allow a proper weighing of feed orts on d 36. The health status of piglets was evaluated and registered daily by trained personnel.

**Table 1:** Composition of the experimental diets fed during the pre-starter (d 1 to 15) and starter (d 16 to 36) phase

	Diets	
Ingredient (g/kg air-dried diet)	Pre-starter	Starter
Corn	316.0	206.0
Wheat	136.0	340.0
Barley	64.0	100.0
Full-fat extruded soybeans	204.0	90.0
Soybean meal (44 % CP)	-	148.0
Soybean meal concentrate (56 % CP)	120.0	25.0
Lactose	86.0	36.0
Calcium carbonate	10.8	8.2
Monocalcium phosphate	12.7	13.0
Salt	3.0	3.0
DL-Methionine	2.2	1.9
L-Lysine-HCl	5.0	5.7
L-Threonine	2.0	2.1
L-Tryptophan	0.3	0.3
Soybean oil	34.0	17.0
Trace elements and vitamin premix^a^	4.0	4.0
Calculated analysis (% or as specified)		
Crude protein (N × 6.25)	19.4	18.5
Digestible energy (MJ/kg)	14.8	13.9
Digestible amino acids^b^		
Lysine	1.38	1.31
Methionine	0.51	0.46
Methionine + cysteine	0.84	0.77
Threonine	0.9	0.85
Tryptophan	0.26	0.23

^a^The mineral and vitamin premix (TEGASA, Barcelona, Spain) provided the following per kg diet: vitamin A 10,000 UI; vitamin D3 2000 UI; vitamin E 25 mg; vitamin B1 1.5 mg; vitamin B2 3.5 mg; vitamin B6 2.4 mg; vitamin B12 20 μg; vitamin K3 1.5 mg; calcium panthotenate 14 mg; nicotinic acid 20 mg; folic acid 0.5 mg; biotin 50 μg; iron 120 mg; iodine 0.75 mg; cobalt 0.6 mg; copper 150 mg; manganese 60 mg; zinc 110 mg; selenium 0.37 mg

^b^Ileal standardized digestibility

### Experimental design and treatments

On d one, 30 min before weaning, animals were randomly assigned to two groups paired by body weight and sex (*n* = 10) that were control (injected i.p. with 4.5 mL of saline solution) and cromolyn (injected i.p. with sodium cromolyn at 20 mg/kg BW; Sigma-Aldrich). The same procedure was repeated at 8 and 16 h relative to weaning time. The dose of sodium cromolyn was established based on a previous study [6].

### Sample collection and analysis for gastrointestinal permeability

Intestinal permeability was assessed in vivo on d 36 after weaning. Eight pigs per treatment were fasted for two h and subsequently sedated with a mixture of xilazine (1.5 mg/kg BW im) and ketamine (11 mg/kg BW im) in order to minimize handling stress. Ten minutes after sedation, animals were intragastrically dosed (gastroduodenal feeding tube, Levin type; VEC Medical) with a marker solution containing 0.5 g mannitol (Sigma-Aldrich, Madrid, Spain) and 0.6 g Co-EDTA [14] dissolved in 15 mL deionized water for a total dose of 1.1 g of marker mixture per pig. Blood samples were collected by jugular venipuncture 1 h after oral infusion of permeability markers into one 5 mL evacuated fluoride/K-oxalate glucose blood collection tubes (BD vacutainer, Madrid, Spain). Plasma mannitol was determined by ultra-high performance liquid-chromatography mass-spectrometry (Xevo G2 TOF, Waters), with an electrospray ionization (ESI) source operating in negative mode. Plasma was injected (5 μL) onto a BEH amide column (2.1 mm × 100 mm, 1.7 μm, Waters). The mobile phases were: A: water + 0.1 % NH_4_OH, and B: methanol + 0.1 % NH_4_OH. Elution conditions, at a flow rate of 0.3 mL/min, were as follows: 90 % B maintained for 2 min, linear gradient from 90 to 60 % in 4 min, and equilibration to initial conditions over 4 min. The MS operating conditions were as follows: source temperature: 120 °C; desolvation temperature: 350 °C; desolvation gas: 900 L/h; cone gas: 10 L/h; capillary voltage: 0.5 kV; cone voltage: 30 V; extraction cone: 4 V; TOF-MS: 80–1000 amu, centroid mode, scan time: 0.3 s. Leucine enkephalin (m/z 554.2615) at a concentration of 2 μg/mL was used as a lockmass for mass accuracy and infused at a flow of 5 μL/min. Chromatograms were processed using Quanlynx software (v 4.1, Waters), individual ions or fragments (m/z 181.07, 161.04, 503.26 for mannitol, and raffinose, respectively) were used for quantification. The concentration of Co in plasma samples was analyzed by atomic absorption spectroscopy [14].

### Tissue collection

On d 36, eight pigs per treatment were sacrificed with captive bolt and exsanguinated. The abdomen was opened, the intestines were removed and the ileum (from the first Peyer’s patch to the ileocecal valve) was dissected. A 10 cm segment was removed from the midsection of the ileum, divided into 5-cm halves, opened longitudinally and flushed with saline. One of these samples was fixed in JB-fix [15] for later MC count. The remaining sample was fixed in 10 % buffered formalin for later histologic determination. A 5-cm sample was collected from the ascendant colon, opened longitudinally and flushed with saline. The mucosa was scraped, stored at −80 °C, and subsequently analyzed by ELISA for cortisol, tumor necrosis factor alpha (TNF-α) (Cusabio Biotech, Hubei, China) and MC tryptase (MCT) (Elabscience, WuHan, China).

### Histological analyses

Samples of ileum were dehydrated and embedded in paraffin, sectioned (~4 μm), and stained with hematoxylin and eosin. Villus height, crypt depth and number of goblet cells in crypts were measured in 10 well-oriented villi and crypts by using a light microscope (Olympus) and a linear ocular micrometer (Olympus). All measurements were performed by the same person who was blinded to the treatments, as described previously [16, 17]. For quantification of MC, ileum samples were fixed in zinc-based fixative [15] and sectioned for immunohistochemistry. Tissue sections were then processed for immunohistochemistry and stained for C-Kit (YR145) (Cell marquee, Rocklin, California). Mast cells were counted at a 40 × magnification using a micrometer grid fitted within an eyepiece. At this magnification, the grid covered a 0.5 mm^2^ area. For each tissue slide, six non-overlapping areas above the muscularis mucosae were counted for the estimation of mucosal MC numbers, which were expressed as cells per mm^2^.

### Statistical analysis

In view of the objectives of the study, data for body weight and average daily gain were analyzed from weaning until the end of nursing phase (d 36). Average daily feed intake (ADFI) and growth efficiency expressed as the ratio between mean ADG and mean ADFI were analyzed from weaning until d 29. For these analyses, the pig was the experimental unit. A mixed-effect model with repeated measures was used in which the effect of pig nested within treatment entered the model as random and the effect of treatment, time (week after weaning) and their two-way interaction were considered fixed.

Results for the recovery of mannitol and Co in plasma samples were analyzed with a mixed-effects model in which the pig nested within the treatment was considered a random variable and treatment as fixed effect. The same model was used to analyze data for ileum histology and concentration of inflammation markers in colon.

Least squares means were separated into significant effects using Tukey’s adjustment. In all cases, the smallest value for the Akaike’s information criterion was used to identify the most appropriate covariance structure and ANOVA was performed using the mixed-model procedure of SAS (release 9.2; SAS Institute). Differences were considered significant when *P* < 0.05, whereas when *P* < 0.05 but ≤ 0.10, differences were considered to indicate a trend toward a significant effect.

## Results

**Fig. 1:**
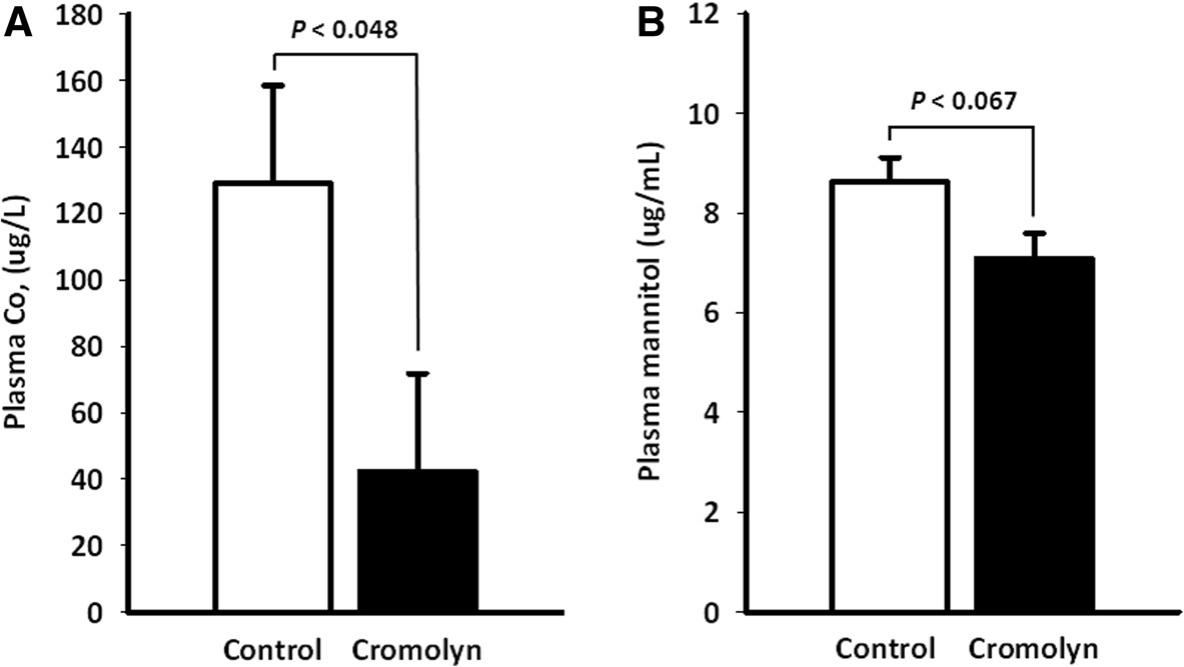
Plasma concentration of Co and mannitol. Co (**a**) and mannitol (**b**) were measured on d 36 after weaning in the plasma of piglets injected i.p. with saline (Control) or 20 mg/kg BW of sodium cromolyn (Cromolyn) at −0.5, 8 and 16 h relative to weaning. Values are least squares means ± SEM; *n* = 8 per treatment

In general, i.p. injections were well tolerated by animals and no adverse side-effects were observed. Two large molecules (i.e., Co-EDTA and mannitol) were used as probes to evaluate the impact of treatments on intestinal permeability. Under the conditions of this study, treating pigs with cromolyn reduced (*P* < 0.048) the plasma concentration of Co by about 70 % (Fig. 1a). Similarly, cromolyn treated pigs exhibited approximately 20 % lower (*P* < 0.067) plasma concentration of mannitol as compared to untreated controls (Fig. 1b). Although not significant (*P* < 0.218), cromolyn-treated pigs had 15 % more granulated MC in the ileum (66.3 ± 0.1 granulated MC per mm^2^) than control animals (51.7 ± 0.1 granulated MC per mm^2^). In addition, piglets in the cromolyn group had similar concentrations of cortisol (3.98 ± 0.54 vs. 2.97 ± 0.58 ng/mg of protein; *P* < 0.201), TNF-α (0.86 ± 0.14 vs. 0.69 ± 0.14 ng/mg of protein; *P* < 0.381) and MCT (69 ± 6 vs. 59 ± 7 pg/mg of protein; *P* < 0.270) in the colonic mucosa than the control counterparts. Treatments did not alter ileal villus height (324 ± 14.1 vs. 322 ± 14.1 μm; *P* < 0.908), crypt depth (231 ± 12.6 vs. 231 ± 12.6 μm; *P* < 0.979) and number of goblet cells per villus (15.9 ± 0.8 vs. 16.9 ± 0.8 goblet cells per villus; *P* < 0.465).

**Fig. 2:**
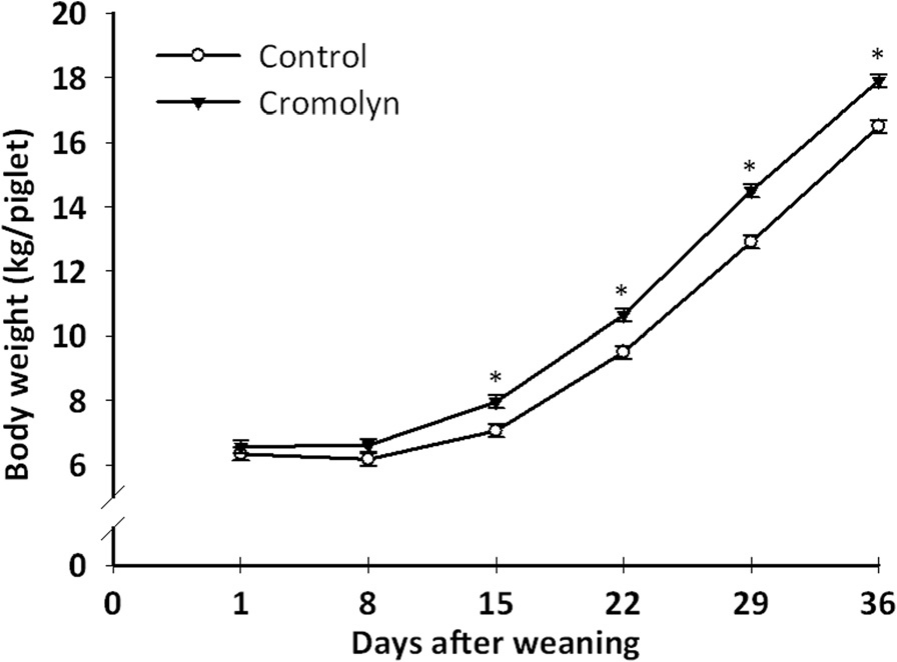
Time course of body weight during the nursing period. Piglets were injected i.p. with saline (Control) or 20 mg/kg BW of sodium cromolyn (Cromolyn) at −0.5, 8 and 16 h relative to weaning. Values are least squares means ± SEM; *n* = 10 per treatment; SEM = 0.2. *P < 0.001

Overall, the amount of feed consumed, growth rate and BW that were achieved throughout the study resemble values observed under current setting of pig production [18]. Remarkably, cromolyn-treated pigs had a significantly higher (*P* < 0.009) feed intake (Table 2), gained more BW (*P* < 0.006; Fig. 2) and grew more efficiently (*P* < 0.042) than pigs in the control group (Table 2).

**Table 2:** Post-weaning performance of piglets injected i.p. with saline (Control) or 20 mg/kg BW of sodium cromolyn (Cromolyn) at −0.5, 8 and 16 h relative to weaning^a^

	Treatments			
Item	Control	Cromolyn	SEM	*P* > F
N	10	10		
Body weight, kg				
Initial body weight, d 1	6.3	6.6	0.2	0.418
Final body weight, d 36	16.5	17.9	0.2	0.002
Average daily gain, g/d	234.7	283.1	11.7	0.006
Average daily feed intake, g/d^b^	313.4	369.0	13.6	0.009
Gain:feed^b^	0.40	0.59	0.2	0.042

^a^Values are least squares means ± SEM

^b^Calculated using data until d 29

## Discussion

In line with published results [6], data reported herein indicate that the administration of a MC-stabilizing agent (cromolyn) to piglets around weaning had beneficial and long-lasting consequences for the integrity of the intestinal mucosa. Specifically, treating piglets with a drug that blocks the weaning-induced activation of the intestinal CRF-MC axis [5, 6] reduced the recovery in plasma of molecules (i.e., Co and mannitol) that enter the bloodstream mainly via paracellular leakage through mucosal tight junctions [8, 14, 19]. These findings demonstrate that cromolyn was effective at preserving intestinal integrity in early-weaned pigs. Noteworthy, the fact that intestinal permeability was assessed 36 d after cromolyn administration suggests that counteracting the intestinal effects of weaning stress with an inhibitor of MC degranulation improved the long-term function of the intestine. Previous work provides compelling evidence indicating that exposure to stress early in life results in sustained dysfunction of the intestinal barrier function through a complex mechanism in which the interaction between neuroimmune factors and intestinal MC plays a fundamental role [20–22]. Barreau and co-workers [23] showed that maternal deprivation during the neonatal phase increased gut mucosal permeability, the number of MC and the expression of cytokines in the colon of rats 12 weeks after exposure to stress. More recently, Smith et al. [11], reported that weaning pigs at 15 to 21 d of age resulted in various signs of paracellular leakage that were still evident at 63 d of age. These effects were attributed to chronic activation of the HPA-axis with a concomitant increase in the lysis of intestinal MC, release of MCT, and alteration of mucosal immune homeostasis. Based on these observations, we speculated that the cromolyn-mediated improvement in intestinal permeability would be paralleled by reduced degranulation of ileal MC and concentration of colonic MCT on d 36 after early weaning. Although changes in these variables were as expected, their magnitude was not large enough to become significant. We cannot rule out, therefore, that in our study cromolyn improved the intestinal integrity of pigs through actions that are unrelated to the inhibition of MC degranulation in the intestine. Indeed, although cromolyn is commonly defined as a “mast cell-stabilizer” that suppresses MC degranulation, previous findings [24, 25] support the idea that the targets of this molecule are not restricted to MC. In order to assess if changes in gut permeability caused by cromolyn treatment were associated with alterations in the architecture of the mucosa we measured the morphology of the ileal epithelium on d 36 after weaning. The dimensions of ileal villi and crypts as well as the number of goblet cells did not differ between control and cromolyn-treated pigs. Even though other intestinal sections were not examined in our study, these results are not surprising because the morphology of the intestinal mucosa appears to recover much faster than its barrier function after weaning-induced damage. Indeed, Hu et al. [26], found that in pigs the architecture of the intestinal epithelium returned to pre-weaning levels 14 d after weaning whereas the gut barrier function was still impaired. Considering that activation of intestinal MC dysregulates the expression and function of tight and adherence junction proteins [27], it is reasonable to suggest that the cromolyn-mediated improvement in gut permeability might have involved functional changes at the level of tight junctional complexes between adjacent mucosal cells. Further mechanistic studies are needed to investigate this possibility at a molecular level. We hypothesized that reducing the negative impact of weaning stress on gut permeability can have long-lasting effects on the mucosal barrier function and thereby enhance animal growth long after weaning. The association between gut hyperpermeability in response to maternal separation and long-term reduction in BW has been already demonstrated in rats [23]. To the best of our knowledge, however, this is the first study to show that reducing the impact of weaning stress on intestinal permeability via treatment with cromolyn is associated with improved performance of pigs fed cereal-based diets. Remarkably, cromolyn treated pigs had a 20 % faster rate of growth and were 1.4 kg heavier than control animals on d 36 after weaning. Provided the magnitude of these effects, confirmation of results under commercial settings of pig production is warranted. Because cromolyn administration also improved the efficiency of feed conversion, the enhancement in BW gain is attributable to factors other than solely increased feed consumption. Noteworthy, enhanced intestinal permeability facilitates the translocation of bacteria from the gut lumen to systemic organs thereby triggering inflammatory and immunological responses [28]. These findings along with data reported herein support the suggestion that reduced gut permeability in the cromolyn-treated pigs might have resulted in a diminished stimulation of the proinflammatory immune response when compared with control animals. If this is true, then it is possible that cromolyn-treated animals grew more efficiently partly by using a larger proportion of absorbed nutrients for growth rather than to support the immune system [10]. Overall, this proof-of-concept study clearly demonstrates that using a MC-stabilizing agent to limit the impact of weaning stress on intestinal permeability resulted in significant improvements in pig performance long after weaning. Interestingly, recent cell-culture studies have shown that various plant bioactives, including some polyphenols, can also inhibit MC degranulation [29]. Therefore, available data provide a rationale for exploring the value of such compounds to mimic the impact of cromolyn on gut permeability and performance of weanling pigs.

## Conclusions

In summary, treating piglets at weaning with the MC-stabilizing agent cromolyn has long-term effects in reducing intestinal permeability deterioration of the gut and enhancing feed consumption, growth, and efficiency of growth long after weaning. Taken together, our results suggest that targeting intestinal MC at weaning may illuminate ways to reduce the magnitude and persistency of the negative impact that weaning stress has on the intestinal integrity, growth, and welfare of pigs.

## Abbreviations

ADFI: average daily feed intake
BW: body weight
Co: cobalt
Co-EDTA: cobalt-ethylenediamine tetraacetic acid
CRF: corticotropin releasing factor
CRF1: corticotropin releasing factor receptor 1
CRF2: corticotropin releasing factor receptor 2
MC: mast cell
MCT: mast cell tryptase
TNF-α: tumor necrosis factor alpha
